# Feeling the Same Strain? A Cross-Sectional Comparison Study of Fathers’ Versus Mothers’ Parenting Stress During the COVID-19 Pandemic

**DOI:** 10.3390/children12081055

**Published:** 2025-08-11

**Authors:** Anna Friedmann, Anne Sophie Wenzel, Katharina Richter, Ina Nehring, Volker Mall

**Affiliations:** 1 Social Pediatrics, School of Medicine and Health, Technical University of Munich, Heiglhofstrasse 69, 81377 Munich, Germany; annesophie.wenzel@tum.de (A.S.W.); katharina.richter@tum.de (K.R.);; 2 German Center for Child and Adolescent Health (DZKJ), Partner Site Munich, Lindwurmstrasse 4, 80337 Munich, Germany; 3 kbo-Kinderzentrum, Heiglhofstrasse 69, 81377 Munich, Germany

**Keywords:** parenting stress, paternal stress, maternal stress, pandemic, family conflict, attachment

## Abstract

**Background/Objectives**: Parenting stress is associated with parent and child mental health problems and has increased since the beginning of COVID-19. Research on paternal parenting stress is sparse—even if family models are changing increasingly with fathers being more strongly involved in caregiving for their children. This study investigated (1) overall parenting stress levels and specific parenting stress subscales in fathers with young children in comparison to mothers and (2) potential influencing factors on fathers’ and mothers’ parenting stress during the pandemic. **Methods**: In a cross-sectional online study, *N* = 368 parents (50.00% fathers) of children (0–3 years) filled out the German version of the Parenting Stress Index (EBI) and answered questions on sociodemographic and pandemic-related factors. **Results**: Fathers were 36.17 years (*SD* = 5.21) and mothers 33.65 years (*SD* = 4.39) old and 67.50% had a high educational background. Children (45.38% female) were 16.34 months (*SD* = 11.66) old. There was no significant group difference between fathers’ and mothers’ overall parenting stress (*p* = 0.39). Parenting stress scores in the attachment subscale were significantly higher in fathers (*p* < 0.001, *r* = 0.19). An increase in family conflicts during the pandemic had the highest impact on both paternal (*ß* = 0.45) and maternal (*ß* = 0.35) parenting stress. **Conclusions**: Parenting stress was equally high for fathers and mothers during the COVID-19 crisis, indicating a levelling of pre-pandemic differences due to pandemic-related factors. Future support measures should focus on reducing family conflicts and on strengthening fathers’ attachment to their child.

## 1. Introduction

As a result of the measures implemented to contain the global COVID-19 pandemic, daily life around the world changed drastically, particularly impacting the wellbeing of families [1,2,3]. One factor closely intertwined with family wellbeing is parenting stress [4], i.e., the burden resulting from the demands of the parenting role. It is usually described as parents’ negative experiences resulting from a perceived disparity between parental responsibilities and available resources [5]. The parents become concerned about their abilities and competencies as a parent and their fulfilment of parental tasks in raising their child.

High or chronic parenting stress can be of enormous clinical significance, since it is associated with a higher psychological vulnerability for parents and children alike [6,7]. For instance, such stress is considered a decisive risk factor for the emergence of mental illnesses in parents, in particular depression [8]. Parenting stress is also associated with less positive parenting behaviors, e.g., more harsh and disciplining behaviors in general [6], and diminished sensitivity and responsiveness [9,10] when taking care of infants and toddlers, specifically reflecting a reduced capacity to promptly and appropriately meet their child’s needs. These factors can have negative effects on the parent-child-relationship [11,12] and interaction [8,13,14], which in turn act as important determinants for adverse child development outcomes and mental health problems across different age groups [6,7,15,16,17].

Consequently, parenting stress may result in a child having a higher risk for excessive crying, sleeping and feeding difficulties [11] and even subsequent health problems at a later age [6]. Parenting stress during middle childhood is positively associated with emotional problems, e.g., anxiety and depressive symptoms as well as behavioral problems [18,19,20]. A recent meta-analysis found that parenting stress correlated significantly with children’s internalizing and externalizing symptoms, specifically during the pandemic [21]. Previous research also revealed parenting stress to be a risk factor for both domestic violence in general and child abuse in particular [22].

A variety of studies have reported an increase in parenting stress since the beginning of the pandemic [23,24,25,26,27,28,29]. Whereas in Germany roughly one third of parents with children aged 0–3 years reported elevated parenting stress before the pandemic [30], a survey conducted in Southern Germany during the pandemic found that up to half of all parents surveyed experienced increased parenting stress levels [26].

Although the pronounced burdens on families and the severe implications for child mental health during the pandemic have raised awareness among clinicians and researchers, the majority of the research in this context has focused on families with school-age children and/or adolescents. Families with very young children have been somewhat overlooked, despite the fact that infants and toddlers are exceptionally vulnerable to psychosocial stressors. During this critical period of rapid development, they rely almost entirely on a secure and responsive parent–child relationship, with their well-being closely intertwined with that of their caregivers [6,7,28,31,32]. Hence, even though infants might have been less directly influenced by structural restrictions to contain the virus (e.g., nursery and school closures), they may have been indirectly affected by the pandemic via their caregiver’s parenting stress and potential impact on their parenting behavior [33,34]. Given the well-established effects of parenting stress on early developmental trajectories and the heightened neurobiological and emotional sensitivity of infants and toddlers, families with young children warrant particular attention in the context of societal crises.

With regard to parenting stress in general, fathers receive almost no attention. The evidence that does exist presents inconsistent findings when comparing fathers’ and mothers’ parenting stress. A meta-analysis of pre-pandemic studies [35] found that mothers reported significantly higher levels of parenting stress than fathers; however, these were caregivers of children with a chronic physical condition. This disparity was often attributed to the fact that mothers typically assume a greater share of familial responsibilities while simultaneously working long hours [36]. Nevertheless, more recent evidence collected after the onset of the COVID-19 pandemic suggests that gender differences in parenting stress have diminished or even disappeared [37], which might more accurately represent the modern living environment of families. Indeed, mothers still seem to play the decisive role in raising children and often spend more time with their children in quantitative terms [38]. In contrast, in the past few years, a discernable change in the paternal role has been observed with fathers more involved in caregiving and child-rearing [38], thereby increasing the probability of fathers facing inter-role conflicts, e.g., career development and parenting responsibilities [39]. This change is also evident in research suggesting that mothers and fathers experience comparable work-family conflicts [40]. Juggling different roles can be associated with conflicting demands of time or energy. In line with the Conservation of Resources Theory [41,42], individuals seek to maintain or expand their available resources. Strain resulting from multiple role expectations may contribute to a perceived lack of necessary resources and the inability to effectively meet role obligations. This perception can provoke a stress response resulting in adverse psychological outcomes like mental tension. Traditionally assigned to the role of breadwinners, fathers now face an expanded set of paternal expectations. This shift, coupled with potential inter-role conflicts, may elevate paternal parenting stress. According to the Daily Hassles Theory [43], seemingly minor daily challenges in managing interactions with the child represent meaningful predictors of parenting stress. As fathers become more engaged in everyday family routines, their exposure to these stressors might also tend to increase.

With the pandemic catalyzing paternal involvement in caregiving [44,45], paternal parenting stress and its determinants must be examined. Nevertheless, during the pandemic, only a negligible number of studies were devoted to parenting stress in fathers of infants and young children and again—comparable to the pre-pandemic evidence—the results were inconsistent. Research indicates that, prior to the onset of the pandemic, fathers reported lower levels of parenting stress compared to mothers; however, paternal stress levels increased as the pandemic progressed [1,27,29,46,47]. Moreover, for the first time, a higher paternal than maternal parenting stress was identified [11], suggesting a potential convergence of parenting stress levels between genders in crisis contexts. In contrast, Abidin et al. [30] found that mothers continued to perceive more parenting stress than fathers during the COVID-19 pandemic. Hence, it remains unclear how the pandemic affected paternal parenting stress and whether fathers’ stress levels differed from those of mothers; however, some evidence suggests that patterns of parenting stress may have shifted, with fathers now approaching or even exceeding the stress levels traditionally reported by mothers.

Moderating factors have barely been investigated in this context. In fact, few studies provide evidence for possible differences with regard to the individual influencing factors of paternal and maternal parenting stress and even less have investigated this topic specifically during the pandemic when multiple new stressors emerged for both parents. When looking at maternal parenting stress during the pandemic and in general, there are a number of sociodemographic and health related influencing factors reported in the literature: lower economic status, financial instability, higher educational attainment, working status, marital status, poor social support and mental health issues could be detected as potential risk factors [48,49]. In contrast, Abidin and colleagues found that financial condition was the only predictor of the parenting stress experienced by fathers [30].

With regard to factors specifically arising from the pandemic, family conflicts emerged as a significant influence on parenting stress levels [33]. However, this has not yet been examined separately for mothers and fathers.

Empirical attention to paternal experience of parenting stress has been limited both before and during the COVID-19 pandemic, a gap that is also evident in our own previous work, despite our research focus on pandemic-related parenting experiences [25,26,33]. Consequently, research on the similarities and differences in parenting stress between mothers and fathers also remains scarce. The lower representation of fathers in stress studies may mask broader trends, and more gender-inclusive research is vital. This is especially pertinent in the early years of life, a period typically characterized by elevated levels of parenting stress [16].

Even when fathers are included in data collection, studies often lack a dedicated theoretical framework on paternal parenting stress, as well as a separate, in-depth analysis and interpretation of paternal data. As a result, they fail to adequately address the possibility that fathers may experience and express parenting stress in ways that differ significantly from mothers—both in terms of intensity and domain-specific patterns. Moreover, research on paternal parenting stress tends to focus primarily on overall stress levels, paying little attention to domain-specific variations. Yet, insight into specific stress patterns and their determinants is crucial for informing targeted prevention efforts or designing tailored intervention programs. According to Abidin’s [5] conceptualization, parenting stress is a multidimensional construct comprising several subdomains, including parental competence, feelings of isolation, attachment, parental health, role restriction, depression, and spouse relationship. A differentiated analysis of these dimensions allows for a more precise understanding of specific stressors fathers might encounter. Understanding stress levels across different domains is critical, as they may substantially influence the quality of the parent–child relationship and interaction patterns between father and child. This is especially critical as these factors are known to be linked to children’s overall health and psychosocial well-being. For instance, in a previous study, the effect of paternal parenting stress on child problem behavior including its mediating role between maternal parenting stress and child problem behavior was discussed to become more relevant with increased paternal engagement in caregiving and play [50].

Within the framework of the family system, the well-being of individual members is deeply interdependent. Elevated stress experienced by one member can have extending effects on the others, thereby influencing the overall family functioning [51,52]. Empirical evidence suggests that paternal parenting stress is associated with reduced involvement in child care, which consequently diminishes both fathers’ and mothers’ perceived quality of family life [53,54]. A comprehensive understanding of responses to crises and related stressors requires a systemic perspective considering all family members alike – not leaving fathers behind.

Within the scope of this cross-sectional study we aimed to compare the overall levels of parenting stress in fathers compared to mothers with children aged 0 to 3 years during the pandemic. Furthermore, we examined potential differences between fathers and mothers across specific parenting stress subscales in order to gain a more nuanced understanding of possible entry points for targeted interventions. In addition, we aimed to identify potential influencing factors on parenting stress among both fathers and mothers, thereby addressing a gap that has remained underexplored in pandemic-related research. In detail, we addressed the following research questions:

1. Did fathers and mothers differ with regard to perceived overall parenting stress levels? Given the emergence of novel stressors during the pandemic and prior findings indicating that paternal parenting stress may have undergone significant change in recent times, we hypothesized that fathers would report parenting stress levels equivalent to those reported by mothers.
2. Did fathers and mothers differ with regard to specific parenting stress subscales?
3. Which sociodemographic and pandemic-related factors might have been predictive for the investigated parenting stress in fathers and mothers?

This paper is organized as follows: the next section outlines the study design, measures, and analysis strategy. Results are then presented in accordance with the underlying research questions and subsequently categorized and discussed in relation to relevant theoretical frameworks and the current state of research. The final section addresses the study’s strengths and limitations, as well as implications for future research and practical application.

## 2. Methods

### 2.1. Study Design

The data of this investigation originated from a large study on intermediate and long-term psychosocial stress factors during high and low incidence phases of the COVID-19 pandemic. More detailed, the authors focussed on perceived pandemic burden, overall parenting stress, and parent and child mental health problems in families with children aged 0–3 years in Southern Germany. The original data (*N* = 2925) were analyzed both cross-sectionally and prospectively longitudinally to provide comprehensive insights into young families during times of crisis [25,33]. However, previous analyses did not explicitly differentiate between fathers’ and mothers’. Therefore, in this paper we present cross-sectional data specifically on parenting stress in fathers compared to mothers collected between February 2021 and March 2022. The study protocol was approved by the Ethics committee of (university, vote no. 322/20 S) and pre-registered in OSF (https://osf.io/search/?q=tksh5&page=1, accessed on 10 February 2025).

### 2.2. Participants

Within the framework of the ‘CoronabaBY study’, all participants were recruited and surveyed via smartphone app “Mein Kinder- und Jugendarzt” (“My pediatrician”; www.monks-aerzte-im-netz.de, accessed on 10 February 2025) which is a well-established communication tool connecting parents with their pediatrician. Study invitation and informed consent form were sent out via app to all eligible patients of the participating pediatricians. All parents of children between 3 months and 3 years who used the app and who understood the German study invitation were eligible to take part (for further details on recruitment see [25,33]. Contrary to the original study, this study applied exclusion criteria: Within the scope of this study, parenting stress was defined as the primary outcome. Therefore, complete EBI data was a prerequisite for inclusion in the analysis. Furthermore, we omitted single parenting as a potential confounder to prevent systematic bias since single parenthood is a risk factor for high parenting stress [55,56]. The rationale for this decision was the fact that all single parents in the large sample were mothers. Hence, data values of these mothers could have distored the results due to the disproportionately large influence of individual cases. At the same time, a sufficiently large sub-sample would not have been available for a meaningful sub-analysis. Of all participants who met these eligibility criteria, *n* = 184 were fathers. To create a comparison sample, the same number of mothers were randomly drawn from the original data set via IBM SPSS Statistics 28, resulting in a total sample of *n* = 368 participants.

### 2.3. Measures

All data were collected by standardized questionnaires via app. Participants were asked questions on general sociodemographic characteristics, perceived pandemic burden, and parenting stress.

Pandemic related restrictions and perceived pandemic burden

By the beginning of the pandemic, no validated tests were available to assess the pandemic induced burdens on families. Therefore, we developed a questionnaire about specific restrictions and perceived burden related to the pandemic. Therein, we posed fourteen questions on the participants’ life and family situation including work and financial status, social contacts and leisure activities, support and care services as well as increased family conflicts during the pandemic. Overall perceived “pandemic burden” derived from the 5-point-answer (from 1 = not at all stressful to 5 = very stressful) to the global question: ‘Taken together, what do you think: How stressful is/was the COVID-19 pandemic for you (please think of measures like social restrictions but also your personal experiences, related worries etc.)?’.

Parenting Stress

To assess parenting stress, the parent domain of the German Version of the ‘Parenting Stress Index’ (“Eltern-Belastungs-Inventar” EBI; Ref. [57] was applied). A recent meta-analysis indicated a particularly high utilitiy of the instrument in the assessment of parental stress [4]. High scores (*T*-value ≥ 60) indicated limited parental resources for upbringing and care for the child and very high scores (*T*-value ≥ 70) implied critically high parenting stress. The parent domain consists of seven subscales, namely ‘health’ (health impairment as a cause or a result of parenting stress), ‘isolation’ (lack of integration in social networks), ‘role restriction’ (perceived limitations as a result of being parent), ‘parental competence’ (parental doubt about their own abilities to manage upbringing and care for their child), ‘attachment’ (emotional relation to the child), ‘depression’ (limited emotional availability within the parent-child-relationship) and ‘spouse related stress’ (due to being a parent). Fathers and mothers indicated their parenting stress experience on a 5-point Likert scale ranging from 1 = *strongly agree* to 5 = *strongly disagree* with a possible score range of 28 to 140. Internal consistency of the parent domain has been proven to be good (*α* = 0.93), and retest reliability after one year has been shown to be 0.87. Correlations with stress indicators and related constructs have resulted in the assumption of test validity [58].

### 2.4. Statistical Analysis

In order to investigate differences in overall parenting stress levels (EBI total score) between fathers and mothers, we conducted a two-tailed *t*-test for independent samples. Since data of EBI subscales were not normally distributed, group comparisons between fathers and mothers were detected using the Mann-Whitney-U-test.

To identify factors related to the parenting stress of fathers and mothers, we conducted two multiple linear regression using backward elimination. Hereby, predictors are iteratively removed based on their contribution to explaining variance in EBI total score for fathers and mothers. This method is particularly suitable for investigating novel and complex contexts with limited knowledge about relevant predictors, specifically the pandemic related predictors.

All described results were based on an alpha level of 5% and Bonferroni correction was applied to control for multiple testing when conducting group comparisons and backwards regressions. Data was analyzed by using IBM SPSS Statistics 28.

## 3. Results

### 3.1. Sample Characteristics

Generally, a large proportion of the participants were from Germany (*N* = 339/92.11%). Moreover, the majority ranked their family’s financial situation before the pandemic to cover arising expenses and to additionally allow cash reserves (94.8%), while few families stated their situation to only cover arising expenses (4.3%) or not cover the costs for the basic living needs (0.8%). Overall, the educational level in this sample was high with 72.28% fathers and 59.34% mothers having at least a university entrance qualification or university degree (*U* = 13954.00, *p* = 0.03, *r* = −0.11). Regarding parenthood, more mothers (58.70%) than fathers (4.35%) were currently on parental leave (*χ*^2^(1) = 125.89, *p* = 0.001, *φ* = 0.585). Overall, 45.38% of the children were girls. Last, in the father sample, 50.00% of the children had siblings while in the mother sample 46.20% had siblings (*χ*^2^(1) = 0.53, *p* = 0.47, *φ* = 0.04). See Table 1 for more descriptive statistics.

### 3.2. Paternal and Maternal Parenting Stress

Fathers (*M* = 76.23) and mothers (*M* = 74.50) did not differ significantly regarding overall parenting stress (*t*(366) = 0.87, *p* = 0.39). In detail, 35.33% fathers and 38.59% mothers experienced high stress (*T* ≥ 60) while 9.78% of the fathers and 7.07% indicated critically high parenting stress scores (*T* ≥ 70). The Mann-Whitney-U-test revealed significant differences for the subscale attachment, with fathers scoring higher than mothers, *U* = 13,137.00, *n*_mothers_ = 184, *n*_fathers_ = 184, *p* < 0.001, *r* = 0.19, indicating a small effect size. The result withstood the Bonferroni correction. See Table 2 for detailed results.

### 3.3. Factors Associated with Paternal and Maternal Parenting Stress

For the multiple backwards regression analyses, the sample size of the paternal group was reduced to *n* = 182 as two fathers did not want to give information about their financial situation. The sample size of the mothers was reduced to *n* = 180 mothers due to single invalid answers regarding the educational level. First, based on theoretical considerations, we determined fixed sociodemographic and specific pandemic related variables as potential influencing factors of parenting stress. In a second step, we identified all variables that correlated significantly with the EBI total score (see Table 3).

Regarding the father sample, child age, the existence of siblings, and all pandemic-specific variables were related to parenting stress. For mothers, child age, parental education, parental leave, parental pandemic burden, as well as pandemic driven increased family conflict, change of care situation, and heightened financial burden correlated with parenting stress.

These variables were then entered as independent variables into two full models for each fathers and mothers with EBI total score as the outcome variable. Increased family conflicts during the pandemic was the strongest associated factor with parenting stress in both fathers (*ß* = 0.45, *p* < 0.001) and mothers (*ß* = 0.35, *p* < 0.001). For mothers, parental education was also significantly associated with parenting stress (*ß* = 0.22, *p* = 0.002). The results remained statistically significant after Bonferroni correction. In total, the regression models explained 27.7% variance of parenting stress in fathers (*F*(3,178) = 24.14, *p* <0.001) and 22.5% variance of parenting stress in mothers (*F*(3,176) = 18.33, *p* < 0.001). Table 4 provides the comprehensive results of the regressions for both paternal and maternal parenting stress.

In order to evaluate detectable effect sizes for individual predictors, a post-hoc power analysis was conducted by using G*Power 3.1.9.7. With a power of 0.80, an alpha level of 0.007 and 182 participants, we were able to detect effect sizes down to *f*^2^ = 0.07 in case of the fathers. Similar, with a power of 0.80, an alpha level of ≤0.01 and 180 participants, effect sizes down to *f*^2^ = 0.07 could have been found in case of the mothers.

## 4. Discussion

This cross-sectional study with *n* = 368 parents of infants and toddlers investigated parenting stress in fathers versus mothers as well as potential influencing factors during the pandemic in Germany. In support of our hypothesis, we found that fathers’ overall parenting stress levels were as pronounced as those of mothers. Additionally, fathers showed significantly higher stress scores in one of the measured parenting stress subscales which related to the attachment to their young child. An increase in family conflicts during the pandemic was most strongly associated with parenting stress in both fathers and mothers.

Our result of equally high overall parenting stress levels in fathers and mothers gains particular importance as fathers have increased their involvement with young children and undertaken a greater share of child-rearing responsibilities since the onset of the pandemic [59,60,61]. According to the Daily Hassles Theory [43], the accumulation of minor daily stressors in parenting can significantly contribute to overall parenting stress. For fathers, increased involvement in daily caregiving routines—especially during challenging times like the pandemic—may have led to greater exposure to these stressors. In addition, the Conservation of resources Theory [41,42] proposes that stress arises when resources such as time, energy, social support, and coping abilities are threatened or lost. During the COVID-19 pandemic, fathers may have faced increased demands—balancing work from home, social isolation, reduced childcare options, and heightened family conflict—that depleted their resources. This loss or threat to resources likely also contributed to heightened parenting stress and feelings of being unable to meet all role expectations effectively. In turn, pronounced parenting stress in fathers has likely negatively influenced the quality of the additional time spent together with their children with possible implications for e.g., father-child-interactions.

The few available results on gender-based differences in parenting stress are somehow inconsistent, limiting the ability to draw generalizable conclusions across studies. Moreover, the majority of these studies were conducted prior to the COVID-19 pandemic, making it difficult to position our results clearly within the existing evidence base. A study by Taubman-Ben-Ari et al. [29] reported higher parenting stress in fathers of infants aged 3–12 months compared to mothers during the pandemic. Notably, this pattern was only observed during the pandemic; prior to its onset, mothers had reported higher levels of parenting stress. Supporting this, several pre-pandemic studies as well as one conducted during the pandemic have generally found higher parenting stress in mothers [48,53,62], who are still typically the primary caregivers. Our finding appears more consistent with literature that, at the very least, does not report higher levels of maternal compared to paternal stress during the pandemic. It is conceivable that the fathers in our sample experienced a significant increase in parenting stress attributable to pandemic-related challenges; however, the cross-sectional nature of our study precludes a direct evaluation of this possibility. Following this line of reasoning, it is likely that mothers also experienced increased stress during the pandemic; however, the lack of significant differences between parents could suggest a relatively stronger increase in paternal stress, potentially reducing pre-pandemic disparities—similar to the observations made by Taubman-Ben-Ari et al. [29]. Nonetheless, this hypothesis requires confirmation through further research, as our data do not allow for testing of these changes.

With mean overall parenting stress levels ranging above the second EBI cut-off, our results are in line with previous studies detecting notably high levels of parenting stress during the pandemic [25,26,27,28,29]. Moreover, the rates of about 45% stressed fathers and mothers in our sample exceed those reported in pre-pandemic German populations, where approximately one third of parents were affected [30], further corroborating an increase in parenting stress attributable to the pandemic. These results indicate a considerable limitation of resources for caring for their child and a definite need for intervention during the pandemic [57]. Bearing in mind the potential damaging effects of parenting stress on parental and child mental health [8,18,19,20], the considerably high number of parents reporting parenting stress is of specific concern since at the same time access to family support services during the pandemic was limited [63]. Hence, families in need might not have been sufficiently reached with unclear consequences for the family strain situation, impact on the parent-child relationship and family wellbeing.

Taking a closer look at potential differences in specific areas of parenting stress, we found that fathers reported significantly more stress with regard to attachment to their child than mothers, i.e., found it more difficult to relate to their child, understand their child’s needs and feel closeness to their child. This type of stress is problematic because secure father–child attachment is crucial for children’s healthy emotional and social development. Reduced paternal sensitivity and responsiveness—often a consequence of attachment-related stress—can contribute to insecure attachment patterns, which are linked to difficulties in emotional regulation, impaired social competence, and an increased risk of behavioral problems [64,65,66].

While to our knowledge there are no specific comparison studies with regard to perceived parenting stress related to attachment, one investigation focused on father-child-attachment during the pandemic and reports a negative impact of pandemic-related psychological distress [67]. Several factors may have contributed to our findings. A first possible explanation is related to the restraints imposed by the pandemic: Pre-pandemic data showed that fathers’ parenting stress is strongly rooted in social isolation [53,62]. Woźniak-Prus et al. [68] and Dikmen-Yildiz [69] found social support to be positively associated with positive father-child relationship experiences during the pandemic. Since social isolation was a global issue during the pandemic, our result of fathers experiencing higher parenting stress with regard to attachment might also be related to this phenomenon. However, fathers and mothers reported being affected to the same extent by pandemic-related social isolation in our study, possibly indicating that fathers are specifically vulnerable to social isolation and, as a consequence, are at risk for a deterioration of attachment. An alternative explanation for the higher attachment-related parenting stress reported by fathers may involve general gender-based differences in attachment processes, which likely predated the pandemic and may influence how attachment challenges are experienced or expressed. This interpretation is supported by earlier findings from Lux et al. [70], who examined parenting stress among parents of children aged 0–3 years in Germany prior to the pandemic using the same instrument as in the present study. They also identified significant differences in attachment-related stress, with fathers being more frequently affected than mothers. Hence, this result suggests that the effect may not be exclusively attributable to the pandemic and rather reflects a gender-specific phenomenon. Findings from a previous study by Portu-Zapirain [71] may explain this observation, showing that mothers tend to establish more secure attachments than fathers, while fathers typically develop more attachment security as the child grows older. Since the children in our sample were infants and toddlers, the latter might have been a relevant factor for our result.

While there are indeed some indications for lower attachment security with fathers compared to mothers in young children, a recent meta-analysis identified that only about 2.1% of the observed variance of security could be explained by parental gender [72]. This raises the possibility of yet an alternative explanation. Societal expectations often shape parental roles in early caregiving in unequal ways. Mothers continue to be widely perceived as the primary attachment figures, especially during the first year of life. Fathers, by contrast, are still frequently positioned as secondary or supporting caregivers, largely due to cultural norms and structural factors such as maternity-focused parental leave systems [73,74]. Gendered expectations have the potential to influence both societal perceptions and actual involvement, possibly limiting fathers’ access to early bonding experiences. Such influences can be especially impactful in the course of a child’s first year of life, a phase recognized as a sensitive period for the development of attachment relationships [75]. In fact, our data shows that only 4% of the fathers were on parental leave during the time of the study, which corroborates the theory of gender-based caregiving-roles. Even with pandemic-related changes such as the shift to working from home, fathers in our study likely still spent less time with their young children than mothers, which may have impacted father–child attachment. Contradictory to this idea, a German study [76] revealed a clear hierarchy of duration of attachment behaviors in infants directed towards mothers over fathers. Strikingly, parental involvement (i.e., hours spent with the child) was not associated with attachment variables. Fathers’ quality of interaction and not quantity of interaction were described to be relevant for infant-father attachment security [9,12,50], and the high levels of overall parenting stress in our sample could have contributed to a lower quality of father-child-interaction with respective impact on attachment.

Taken together, fathers of young children might generally experience more difficulty in establishing secure attachment relationships during early parenthood, potentially due to differing caregiving roles or societal expectations. However, we cannot determine the underlying cause for the difference in attachment-related parenting stress between fathers and mothers on the basis of our data. Further research is needed to explore the mechanisms underlying these differences and to examine how external stressors, such as a global health crisis, may have exacerbated or reinforced pre-existing patterns.

Finally, we explored potential influencing factors for both paternal and maternal overall parenting stress separately. In both groups, pandemic-induced increases in family conflict were the most strongly associated factor, demonstrating a medium effect size and supported by the post-hoc analysis as a robust finding. For the father sample, pandemic-related increases in family conflict were the only significant factor in the final model, suggesting a stronger impact of the surveyed pandemic-related stressors compared to sociodemographic variables on paternal parenting stress. Considering more than 20% of the fathers and mothers experiencing an increase in family conflicts during the COVID-19 pandemic [77,78] and families with younger children particularly being affected [78], we assume the interdependence of the increase of family conflicts and parenting stress might have been crucial for the well-being of families during the pandemic. Our findings are consistent with pre-pandemic and pandemic research among families with children aged 0–3: Frequent family conflicts predicted parenting stress [26,79]. More precisely, family conflicts mediated the linkage between children’s behavior problems and later parenting stress [79].

While we only found one significant predictor in the final model for fathers’ parenting stress, the educational level was an additional potential influencing factor for parenting stress amongst mothers, with a higher educational level predicting higher parenting stress. However, the effect size was small. This result stands in contrast with pre-pandemic findings [30,80] but is align with pandemic-related studies [48,81]. For instance, Moghanibashi-Mansourieh [82] showed that individuals with higher levels of education were more anxious with regard to the pandemic and parents with higher anxiety levels are more likely to experience parenting stress [53]. Future research might determine why education was not a contributing factor to paternal parenting stress but was linked to maternal stress experience.

In sum, the elevated parenting stress found among both mothers and fathers in our sample may have significant long-term implications for families. Prolonged or chronic parenting stress in early parenthood can have lasting effects on both parent and child well-being. Emerging evidence suggests that parenting stress has remained high in families with young children even after the acute phase of the pandemic [83]. These findings highlight the need for accessible parenting support programs focused on stress reduction and strengthening father–child attachment.

Several limitations exist in our study that warrant consideration when generalizing the study’s findings. The sample consisted of financially relatively well-off families with good education and German background which may have resulted in less attention to other factors that are of relevance for less affluent families and limits the degree of applicability to the general population. Moreover, we did not collect data of couples but separately for mothers and fathers of different families, which leads to a limited overall generalizability. Future studies have the potential to advance the scholarly understanding by including populations with greater socioeconomic diversity and by undertaking within-family comparative analyses of parents. Following from this, evidence shows fathers’ participation is more likely skewed towards those actively involved in caregiving [84]. This could also be true for participation of fathers in our sample and might have influenced the results. Since single parenthood is a significant factor influencing parenting stress and all single parents in the main study’s dataset were mothers, we excluded these individuals to avoid bias in our results. Including only single mothers without comparable single fathers could have introduced confounding effects related to the unique challenges of single parenting. By focusing on partnered parents, we ensured a more balanced comparison between mothers and fathers, improving the internal validity of our gender-related findings. However, this limits the generalizability to single-parent families, which future studies should examine.

Last, analyses were conducted under the assumption that the experience of the pandemic had crucial influence on the well-being of parents. Nonetheless, on account of the use of a cross-sectional design, the results should not be understood as causal. Additionally, we cannot provide insights on gender-related parenting stress trajectories during the pandemic due to the study design. Gaining knowledge with regard to whether stress patterns persist, shift, or attenuate over time depending on gender would have been relevant for e.g., the development of targeted interventions. Further longitudinal research is needed to achieve a deeper understanding of underlying pathways and to evaluate the potential consequences of high parenting stress in both mothers and fathers of infants during the pandemic for long-term healthy development, parent-child-relationships and family wellbeing. As this study was based on a secondary data analysis, it was not possible to account for additional relevant confounding variables, such as caregiving responsibilities, type of employment, or the availability of extended family support, which might have been relevant for parenting stress. Nevertheless, there are hardly any comparable studies with a focus on fathers and beyond of young children during the pandemic. Moreover, our main outcome assessment is based on a standardized questionnaire with complete data for each EBI item by all included fathers and mothers to guarantee the analysis of the overall parenting stress. In contrast to other studies, we were able to collect data over a long period of time under the conditions of the pandemic. In consideration of the increasing contribution of fathers in care work, this study provides a significant insight into paternal parenting stress and corresponding potential influencing factors.

## 5. Conclusions

The study supports the assumption that fathers’ parenting stress intensified during the pandemic, equaling that of mothers. In this context, both fathers and mothers reached alarming stress levels. Our data further indicate a particular vulnerability among fathers regarding attachment-related parenting stress. Increased family conflict during the pandemic was associated with overall parenting stress in both fathers and mothers.

Consequently, our findings highlight the urgent need for targeted interventions in early parenthood in the aftermath of the pandemic. Within the healthcare system, routine screenings for parenting stress in pediatric and primary care settings could help identify at-risk families, enabling timely referrals to counseling, parenting programs, or stress management—thereby preventing a potential escalation of parenting stress into mental health issues such as parental burnout. Support offers could benefit from components that foster secure attachment relationships, support emotional regulation, and provide practical stress coping strategies. They should also promote constructive conflict management strategies that can strengthen the family climate. Parenting programs and mental health services should be specifically tailored to fit both fathers and mothers and account for the unique stressors experienced by each parent. Subsequently, integrated, gender-sensitive approaches may enhance both the engagement and effectiveness. More broadly, policies such as improved parental leave, flexible work arrangements, and implementation of community support networks could also help mitigate parenting stress and promote family wellbeing in the long term.

This study’s generalizability is limited by a socioeconomically homogeneous sample, separate data collection from mothers and fathers of different families, reliance on cross-sectional data, and the lack of control for some contextual variables such as caregiving roles and social support. Given these limitations and the still-emerging research on paternal parenting stress, further investigations are needed to better understand and support fathers as they face increasing parenting demands. Future studies should aim to disentangle gender- from role-related parenting stress patterns and attachment, while also accounting for societal norms and expectations regarding parenting. Building on our findings, research that recruits socioeconomically diverse samples will enhance the generalizability and causal interpretation of parenting stress patterns. Finally, since elevated parenting stress may persist beyond crises such as the COVID-19 pandemic, longitudinal studies are essential to investigate the long-term effects on child and family well-being, as well as to evaluate sustained and accessible support measures and resources.

## Figures and Tables

**Table 1:** Sample Characteristics.

			Fathers		Mothers		
	*N*	Range	*M*	*SD*	*M*	*SD*	*p*
Child Age [months]	368	1–43	16.53	11.61	16.16	11.73	n.s. ^a^
Child Pandemic Burden	368	0–4	1.88	1.24	1.99	1.31	n.s. ^b^
Parental Age [years]	368	21–55	36.17	5.21	33.65	4.39	<0.001 ^a^
Parental Pandemic Burden	368	0–4	2.51	0.97	2.73	0.92	0.04 ^b^
Pandemic Family Conflict	368	0–4	1.28	1.39	1.12	1.14	n.s. ^b^
Pandemic Change of Care Situation	368	0–4	1.55	1.46	1.25	1.33	n.s. ^b^
Pandemic Financial Burden	357	0–4	2.63	0.66	2.71	0.63	n.s. ^b^

*Note*. ^a^: two-tailed *t*-test, ^b^: two-tailed Mann-Whitney-U-Test.

**Table 2:** Paternal and Maternal Parenting Stress Subscales (EBI).

			Fathers		Mothers		
	*n*	Range	*M*	*SD*	*M*	*SD*	*p*
Total score	368	28–127	76.29	18.70	74.50	20.83	n.s. ^a^
*Subscales* Social Isolation	368	4–20	10.49	3.80	9.99	3.90	n.s. ^b^
Attachment	368	4–19	9.91	3.39	8.63	3.24	<0.001 ^b^
Health	368	4–20	11.10	4.07	10.80	4.13	n.s. ^b^
Role Restriction	368	4–20	11.61	3.75	11.49	4.30	n.s. ^b^
Depression	368	4–20	11.51	3.41	11.93	3.93	n.s. ^b^
Competence	368	4–20	9.84	3.54	9.77	4.06	n.s. ^b^
Partner Relationship	368	4–20	11.83	3.50	11.84	3.85	n.s. ^b^

*Note*. ^a^: two-tailed *t*-test, ^b^: two-tailed Mann-Whitney-U-Test.

**Table 3:** Correlations between study variables for both mother and fathers.

	Fathers										
	1	2	3	4	5	6	7	8	9	10	11
(1) EBI total score	1										
(2) Parental Age [years]	0.07	1									
(3) Child Age [months]	0.23 **	0.25 ***	1								
(4) Siblings	−0.18 **	0.31 ***	0.25 ***	1							
(5) Parental Education	0.02	0.11	−0.03	−0.13	1						
(6) Parental Leave	0.08	0.05	0.06	0.21 **	0.03	1					
(7) Child Pandemic Burden	0.26 ***	0.13	0.25 ***	0.47 ***	−0.23 **	−0.02	1				
(8) Parental Pandemic Burden	0.24 ***	0.05	0.10	0.21 **	−0.12	0.07	0.45 ***	1			
(9) Pandemic Family Conflict	0.53 ***	0.04	0.21 **	0.26 ***	0.01	0.11	0.37 ***	0.38 ***	1		
(10) Pandemic Care Situation	0.32 ***	0.26 ***	0.23 **	0.44 ***	−0.06	0.02	0.50 ***	0.18 *	0.37 ***	1	
(11) Pandemic Financial Burden	−0.20 **	−0.02	−0.05	−0.20 **	0.27 ***	0.02	−0.29 ***	−0.26 ***	−0.29 ***	−0.23 **	1
	**Mothers**										
	1	2	3	4	5	6	7	8	9	10	11
(1) EBI total score	1										
(2) Parental Age [years]	0.02	1									
(3) Child Age [months]	0.25 ***	0.29 ***	1								
(4) Siblings	0.02	0.23 **	0.24 ***	1							
(5) Parental Education	0.24 ***	0.18 *	0.12	−0.10	1						
(6) Parental Leave	−0.16 *	−0.13	0.57 ***	−0.09	−0.04	1					
(7) Child Pandemic Burden	0.08	0.08	0.15 *	0.32 ***	−0.26 ***	−0.14	1				
(8) Parental Pandemic Burden	0.23 **	−0.05	0.01	0.08	0.05	−0.02	0.48 ***	1			
(9) Pandemic Family Conflict	0.45 ***	0.18 *	0.24 ***	0.16 *	0.19 **	−0.19 *	0.28 ***	0.38 ***	1		
(10) Pandemic Care Situation	0.11	0.25 ***	0.34 ***	0.35 ***	0.16 *	−0.24 ***	0.36 ***	0.23 **	0.31 ***	1	
(11) Pandemic Financial Burden	−0.08	0.05	0.06	0.03	0.13	−0.07	−0.22 **	−0.16 *	−0.25 ***	0.00	1

*Note.* The marked area refers to metrically scaled variables (Pearson Correlation coefficients) whereas the unmarked area indicates correlations computed with at least one ordinally scaled variable (Spearman Correlation coefficients). * *p* ≤ 0.05, ** *p* ≤ 0.01, *** *p* ≤ 0.001, two-tailed.

**Table 4:** Backwards regression analysis to predict EBI total score for both fathers and mothers.

	*F*	*p*	*Adj.* *R^2^*	*ΔR^2^*	*B*	*ß*	*p*	*CI* (95%)	
								*LL*	*UL*
*n* = 182	**Fathers**								
**Step 1**	10.19	<0.001	0.26	0.29					
Child Age					0.09	0.12	0.076	−0.01	0.19
Siblings					0.47	0.03	0.720	−2.13	3.08
Child Pandemic Burden					−0.13	−0.02	0.830	−1.32	1.06
Parental Pandemic Burden					0.34	0.03	0.649	−1.02	1.63
Pandemic Family Conflict					3.38	0.44	<0.001	2.25	4.50
Pandemic Care Situation					0.78	0.13	0.092	−0.13	1.70
Pandemic Financial Burden					−0.13	−0.01	0.884	−1.93	1.67
**Step 2**	11.96	<0.001	0.27	<0.001					
Child Age					0.09	0.12	0.076	−0.01	0.19
Siblings					0.47	0.03	0.724	−2.13	3.06
Child Pandemic Burden					−0.12	−0.02	0.840	−1.30	1.06
Parental Pandemic Burden					0.32	0.04	0.632	−0.99	1.63
Pandemic Family Conflict					3.39	0.44	<0.001	2.30	4.50
Pandemic Care Situation					0.79	0.13	0.089	−0.12	1.70
**Step 3**	14.42	<0.001	0.27	<0.001					
Child Age					0.09	0.12	0.077	−0.01	0.19
Siblings					0.55	0.03	0.661	−1.91	3.01
Parental Pandemic Burden					0.27	0.03	0.662	−0.95	1.49
Pandemic Family Conflict					3.38	0.44	<0.001	2.30	4.48
Pandemic Care Situation					0.76	0.13	0.084	−0.10	1.62
**Step 4**	18.06	<0.001	0.27	−0.00					
Child Age					0.09	0.12	0.075	−0.01	0.19
Siblings					0.48	0.03	0.697	−1.95	2.92
Pandemic Family Conflict					3.46	0.45	<0.001	2.43	4.49
Pandemic Care Situation					0.76	0.13	0.084	−0.10	1.61
**Step 5**	24.14	<0.001	0.28	−0.00					
Child Age					0.09	0.11	0.082	−0.01	0.18
Pandemic Family Conflict					3.45	0.45	<0.001	2.42	4.47
Pandemic Care Situation					0.70	0.12	0.088	−0.10	1.50
*n =* 180	**Mothers**								
**Step 1**	11.12	<0.001	0.22	0.24					
Child Age					0.12	0.14	0.095	−0.02	2.67
Parental Education					2.14	0.22	0.002	0.81	4.47
Parental Pandemic Burden					0.67	0.06	0.405	−0.91	2.25
Pandemic Family Conflict					2.96	0.33	<0.001	1.62	4.30
Parental Leave					0.81	0.04	0.638	−2.57	4.19
**Step 2**	13.91	<0.001	0.22	−0.00					
Child Age					0.10	0.12	0.089	−0.02	0.22
Parental Education					2.16	0.22	0.001	0.84	3.48
Parental Pandemic Burden					0.68	0.06	0.397	−0.90	2.25
Pandemic Family Conflict					2.94	0.33	<0.001	1.60	4.27
**Step 3**	18.33	<0.001	0.23	−0.00					
Child Age					0.10	0.11	0.099	−0.02	0.22
Parental Education					2.15	0.22	0.002	0.83	3.47
Pandemic Family Conflict					3.16	0.35	<0.001	1.92	4.39

***Note***. CI = confidence interval; LL = lower limit; UL = upper limit. ß = 0.10–0.29 (small effect size), ß = 0.30–0.49 (medium effect size), ß ≤ 0.50 (large effect size).

## Data Availability

Data have not been shared because depositing data to a public access site was not part of the consent process. Materials can be provided by contacting the corresponding author.
